# Deconvolution Tactics and Normalization in Renal Spatial Transcriptomics

**DOI:** 10.3389/fphys.2021.812947

**Published:** 2022-01-13

**Authors:** Ricardo Melo Ferreira, Benjamin J. Freije, Michael T. Eadon

**Affiliations:** Division of Nephrology, Indiana University School of Medicine, Indianapolis, ID, United States

**Keywords:** spatial transcriptomics, visium gene expression, single nuclear RNA sequencing, nephron, acute kidney injury, biopsy specimen

## Abstract

The kidney is composed of heterogeneous groups of epithelial, endothelial, immune, and stromal cells, all in close anatomic proximity. Spatial transcriptomic technologies allow the interrogation of *in situ* expression signatures in health and disease, overlaid upon a histologic image. However, some spatial gene expression platforms have not yet reached single-cell resolution. As such, deconvolution of spatial transcriptomic spots is important to understand the proportion of cell signature arising from these varied cell types in each spot. This article reviews the various deconvolution strategies discussed in the 2021 Indiana O’Brien Center for Microscopy workshop. The unique features of Seurat transfer score methodology, SPOTlight, Robust Cell Type Decomposition, and BayesSpace are reviewed. The application of normalization and batch effect correction across spatial transcriptomic samples is also discussed.

## Introduction

Spatial transcriptomics was selected as Nature’s Method of the year in 2020 (Marx, 2021). As presented at the 2021 O’Brien Center for Microscopy workshop, Spatial Transcriptomics (ST) represents a powerful tool to reveal *in situ* transcript expression associated with histopathologic features. Countless examples of ST in the development of human tissue atlases are available, identifying key features in breast cancer (Wu et al., 2021), Alzheimer’s progression (Navarro et al., 2020), and cardiovascular development (Asp et al., 2019). In the kidney, ST has been applied to understand the regional expression differences in sepsis and ischemia reperfusion injury murine models (Janosevic et al., 2021; Melo Ferreira et al., 2021). A significant limitation of some ST techniques is their resolution. For example, Visium Spatial Gene Expression (VSGE) platform has a spot size of 55 μm and resolution of 100 μm, which invariably encompasses multiple cells within a single spot. Cell atlases of the kidney now include annotation of over 100 different cell types and cell states from a diverse pool of epithelial, stromal, and endothelial cells (Lake et al., 2021). These classes of cell types align very well with the underlying histology of the human kidney (Melo Ferreira et al., 2021). The 55 μm spot size is approximately the size of a cross sectional proximal tubule and will often capture elements of the signature from neighboring peritubular capillaries, dendritic cells, and other stromal cells. To better appreciate the contribution of less represented cell types to a spot’s signature, strategies can be employed to deconvolute the proportion of signature arising in a spot using single cell and single nuclear RNA sequencing (sc/snRNAseq) cluster identities. This brief review outlines the unique features of several deconvolution tactics discussed in the O’Brien center workshop. Normalization and batch effect correction across ST samples are also discussed.

## Deconvolution Techniques

**Figure 1:**
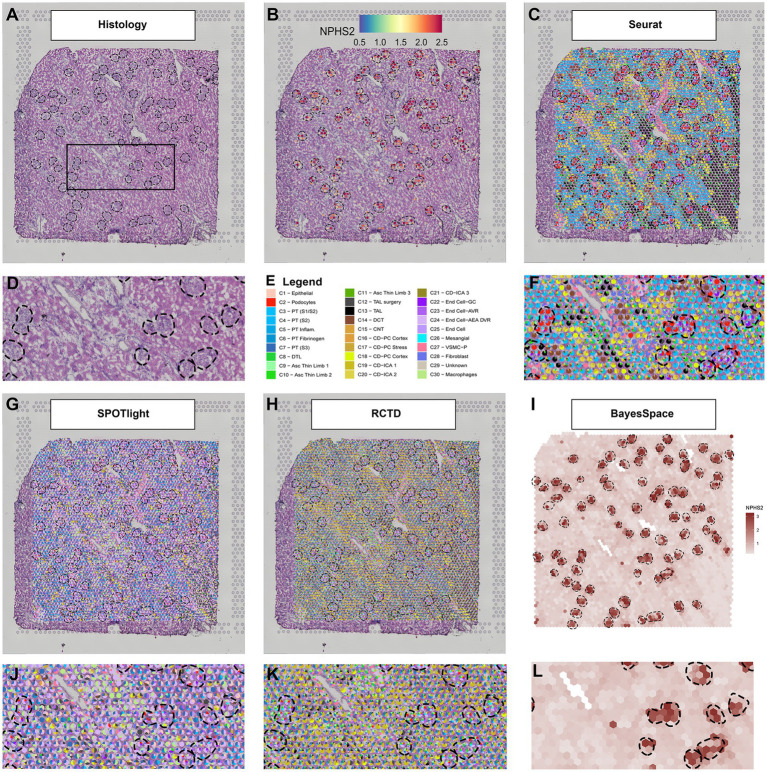
Deconvolution techniques in spatial transcriptomics. **(A)** H + E image of a human nephrectomy sample, **(B)** *NPHS2* (podocin gene) expression localizes over glomeruli. **(C)** Seurat deconvolution in the same nephrectomy field. **(D)** Magnified field of H + E image. **(E)** Cell type identity legend. **(F)** Zoomed image of the Seurat deconvolution. **(G)** SPOTlight deconvolution. **(H)** Robust cell type decomposition deconvolution. **(I)** BayesSpace Deconvolution. **(J)** Zoomed field of SPOTlight deconvolution. **(K)** Zoomed field of RCTD. **(L)** Zoomed field of BayesSpace. Each spot is 55 μm in diameter.

An example of the output from four deconvolution techniques is provided in Figure 1. A human deceased donor nephrectomy without evidence of kidney disease was scored to fit the capture area of the Visium slide and a high-resolution image of the Hematoxylin and Eosin (H + E) stained tissue was taken with a Keyence BZ-X810 microscope as mosaics of 10× fields and stitched (1A). The histological image of the nephrectomy had the glomeruli identified and a magnified region is provided. The tissue was permeabilized and mRNA was captured in barcoded spots that allowed downstream informatic localization of each read after sequencing. The resulting expression of *NPHS2* (1B) is concentrated over the outlined glomeruli. Due to its 55 μm diameter, each spot generally covered multiple cell types. Below we present four methods designed to deconvolute the constituents of each spot. As a reference, we use a publicly available human kidney snRNAseq dataset (Lake et al., 2019).

## Seurat

Seurat is a popular tool to process sc/snRNAseq and ST data, with extensive documentation and support from several other analysis packages. In its version 3, Seurat introduced an anchor methodology to integrate multiple datasets (Stuart et al., 2019) that was further adapted to transfer single-cell cluster information to ST. This procedure results in a transfer score and the highest score can be used to label the spots. Alternatively, the relative scores can be displayed in a pie chart including the components of cell signature arising from multiple single-cell clusters (1C). In the example provided, the more prominent scores in the glomeruli spots are derived from the podocyte, glomerular capillary endothelial cell, and mesangial cell clusters. In the magnified region, glomeruli are surrounded by enriched areas of various proximal tubule (PT), distal convoluted tubule (DCT), and collecting duct (CD) cell signatures, as expected. The Seurat pipeline for ST analysis is still under development. For example, version 3.6.3 presents remarkable agreement between the snRNAseq cell type signatures and the underlying kidney histopathology, with the expected proportion of cell signature correlating strongly with the quantitative proportions of cells in the histology (Lake et al., 2021; Melo Ferreira et al., 2021). However, we have noted reduced alignment between the histology and snRNAseq cluster identity in Seurat version 4.

## Spotlight

The SPOTlight deconvolution method uses a negative matrix factorization regression algorithm to define topics as distributions of gene expression across cell types in the reference dataset. Those topics are then used to define the cell type composition of spots and is directly related to cell type expression profiles (Elosua-Bayes et al., 2021). The results are given in proportions, which are easily interpretable. Its source code was adapted to display deconvolution results in three of the four methods discussed in this review. In the example nephrectomy (Figure 1G), endothelial cell type signatures, both afferent and efferent arterioles (AEA) and descending vasa recta (DVR), dominated the mapping in the tissue, including spots overlaying glomerular histology. Other expected cell types, such as podocytes, glomerular capillary endothelial cells, and mesangial cells, contributed to the cell signature in a disproportionately smaller degree than the underlying histopathology would suggest. The macrophage signature also contributed to a large proportion of spots in the tubulointerstitium and across the tissue. This methodology may require further adjustment of parameters for the kidney because so many distinct functional structures (glomerulus, PT, DCT, etc.) are located in close proximity to each other. In the example provided, the technique identified a greater proportion of signature from components that are broadly distributed across the whole kidney (like endothelial cells and macrophages) rather than specific localized cell types (like podocytes or DCT cells). However, SPOTlight provides several tools to evaluate and correct the deconvolution method, and with adjustments, the alignment between the histology and snRNAseq cluster identity can be improved.

## Robust Cell Type Decomposition

Robust cell type decomposition (RCTD; Cable et al., 2021) also defines cell type transcriptomic profiles. This approach considers each spot as a mixture of cells and fits a statistical model to determine each spot composition. Our results (Figure 1H) show a large contribution of endothelial cell types [afferent arteriole (AEA), DVR] in the glomerular spots, with podocytes and the glomerular capillary endothelial cells represented to a lesser extent. A very minor contribution is observed from the mesangial cell cluster. Across the nephrectomy, the contribution of proximal tubules to the signature is disproportionately low compared to the histology and the collecting duct signature is minor dominant. RCTD potentially performs better on other ST technologies (like slideSEQ) where more than two cell types are rarely seen underlying a single spot (Stickels et al., 2021). The deconvolution method in the algorithm is designed to report the confidence of doublets or singlets underlying a spot.

## Bayesspace

The BayesSpace method approaches deconvolution differently than the three previous examples. Instead of deconvoluting the cell types of each spot, it aims to increase the spatial resolution by interpolating the expression between spots (Zhao et al., 2021). This method applies an unsupervised clustering algorithm to the data that requires an *a priori* definition of the number of clusters. It then interpolates expression and defines those clusters in higher resolution. As an example, we present the interpolated expression of *NPHS2* (Figure 1I). The expression interpolation could be useful to predict gene expression in smaller structures. The interpolated clusters would be an excellent target to apply cell type decomposition algorithms. However, the Seurat, SPOTlight, and RCTD methods are not currently compatible with BayesSpace because these methods would require either raw counts or method-specific normalized expression to integrate with BayesSpace.

## Normalization and Batch Correction

In an effort to create a spatially anchored atlas of the kidney, analysis of multiple ST samples is invariably expected. On the VSGE platform, four samples are run in parallel on a single slide which can lead to batch effects between slides. Furthermore, variations in sample quality can lead to downstream differences in the number of reads mapped to exons in each spot. Differences in permeabilization time, RNA quality, tissue thickness, and tissue sources all contribute to the between sample variability. In sc/snRNAseq, technical variations are reduced through normalization and batch correction, typically through programs, such as ComBat-seq, Harmony, Liger, and Seurat 3 (Welch et al., 2018; Korsunsky et al., 2019; Stuart et al., 2019; Zhang et al., 2020).

**Figure 2:**
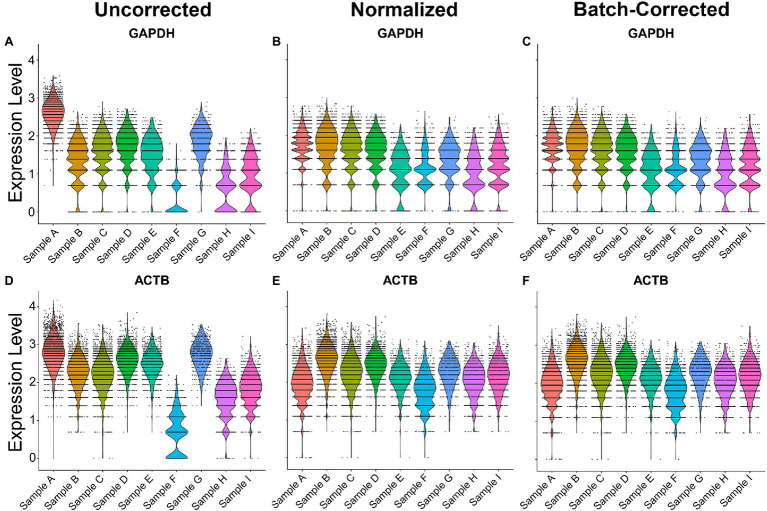
Normalization and batch correction of spatial transcriptomic samples. **(A)** Uncorrected *GAPDH* expression across samples. **(B)** Normalized *GAPDH* expression with SCTransform. **(C)** Normalization and batch correction were performed by adding a batch as a variable in SCTransform. **(D)** Uncorrected expression of *ACTB.* **(E)** Normalized expression of *ACTB*. **(F)** Batch-corrected expression of *ACTB*. Sample A is batch 1, samples B–E are batch 2, and samples F–I are batch 3.

To normalize and batch correct ST data, we provide an example of the regularized negative binomial regression normalization technique, known as SCTransform (Hafemeister and Satija, 2019). To showcase its utility in human samples, nine samples across 3 batches were merged *via* the merge function and normalized or batch-corrected *via* SCTransform. Without normalization and batch correction, the ST samples exhibited inconsistent expression of the house-keeping genes *ACTB* and *GAPDH*, demonstrating a potential need for normalization when comparing across samples (Figure 2). Normalization with SCTransform yielded more comparable gene expression of *ACTB* and *GAPDH* across samples. The inclusion of batch as a variable in the SCTransform tool revealed only a minor additional improvement in gene expression alignment compared to normalization without a distinct batch effect variable. This indicates that technical variation in our samples can be modeled by sequencing depth alone. Together, these results suggest SCTransform may be a useful tool for removing intersample technical variation in ST datasets.

## Conclusion

This brief review presents the result of four common deconvolution techniques and a common normalization procedure applied to the human kidney, as discussed in the 2021 O’Brien Center for Microscopy workshop. The VSGE platform facilitates direct mapping of expression signatures over a H + E stained image. While every organ is different, the kidney has many small, functionally distinct parts of the nephron, all lying in close proximity to each other. Thus, deconvolution of larger spot sizes is essential to mapping the ST signatures. Further, normalization and batch effect correction are important because an atlas must integrate data from multiple sources. The results of the deconvolution methods varied considerably, even when interrogating the same field of tissue. Some methods yielded signatures approximating the underlying histology and others emphasized less abundant cell types. No judgment has been made as to whether the cell type proportions of a spot signature *should* parallel the histologic cell type distribution or whether certain cell types may have an outsized influence on the signature. Differences may arise from how each technique handles cell type heterogeneity or variation in expression. Further, performance can vary based on the fine-tuning of parameters; thus, this review is not intended to compare of each method’s value. Instead, it merely provides an example of the diversity of possible results, depending on the approach selected.

## Author Contributions

All authors listed have made a substantial, direct, and intellectual contribution to the work, and approved it for publication.

## Funding

This work was supported by NIH/NIDDK K08DK107864 (ME); Indiana Grand Challenge Precision Health fund (RM); and the Indiana Center for Biological Microscopy (NIH-NIDDK P30DK079312).

## Conflict of Interest

The authors declare that the research was conducted in the absence of any commercial or financial relationships that could be construed as a potential conflict of interest.

## Publisher’s Note

All claims expressed in this article are solely those of the authors and do not necessarily represent those of their affiliated organizations, or those of the publisher, the editors and the reviewers. Any product that may be evaluated in this article, or claim that may be made by its manufacturer, is not guaranteed or endorsed by the publisher.
